# Transcriptional Studies on *Trypanosoma cruzi* – Host Cell Interactions: A Complex Puzzle of Variables

**DOI:** 10.3389/fcimb.2021.692134

**Published:** 2021-06-17

**Authors:** María Gabriela Libisch, Natalia Rego, Carlos Robello

**Affiliations:** ^1^ Laboratorio de Interacciones Hospedero Patógeno-UBM, Institut Pasteur de Montevideo, Montevideo, Uruguay; ^2^ Unidad de Bioinformática, Institut Pasteur de Montevideo, Montevideo, Uruguay; ^3^ Departamento de Bioquímica, Facultad de Medicina, Universidad de la República, Montevideo, Uruguay

**Keywords:** *Trypanosoma cruzi*, Chagas disease, transcriptomics, respiratory chain, host-pathogen, Chagas cardiomyopathy, experimental variables

## Abstract

Chagas Disease, caused by the protozoan parasite *Trypanosoma cruzi*, affects nearly eight million people in the world. *T. cruzi* is a complex taxon represented by different strains with particular characteristics, and it has the ability to infect and interact with almost any nucleated cell. The *T. cruzi*-host cell interactions will trigger molecular signaling cascades in the host cell that will depend on the particular cell type and *T. cruzi* strain, and also on many different experimental variables. In this review we collect data from multiple transcriptomic and functional studies performed in different infection models, in order to highlight key differences between works that in our opinion should be addressed when comparing and discussing results. In particular, we focus on changes in the respiratory chain and oxidative phosphorylation of host cells in response to infection, which depends on the experimental model of *T. cruzi* infection. Finally, we also discuss host cell responses which reiterate independently of the strain, cell type and experimental conditions.

## Introduction

Chagas disease, discovered more than 100 years ago by the Brazilian physician Carlos Chagas, affects nearly eight million people mostly in rural areas of Latin America (Schmunis and Yadon, 2010). It is considered a neglected tropical disease (NTD) by the World Health Organization (WHO), affecting the very poorest in society, which receive insufficient treatments and preventive measures. Its causative agent, the protozoan parasite *Trypanosoma cruzi*, has a complex life cycle alternating between an hematophagous triatomine insect and a mammalian host, exhibiting different forms during its life cycle (Kollien and Schaub, 2000; Parodi-Talice et al., 2007). During a blood meal, the insect ingests from the host the infective and non-replicative form trypomastigotes. They migrate to the vector´s digestive tract where they transform into epimastigotes, a replicative and non-infective form, which in the hindgut differentiate into the infective and non-replicative metacyclic trypomastigotes. These forms are excreted with faeces of the insect vector and enter the mammalian hosts through the injured skin or the mucosa. Once the parasite reaches the bloodstream it can spread easily, because it has the ability to infect almost any nucleated cell. Intracellularly, the parasite undergoes differentiation into amastigotes, a replicative and also infective (Mortara et al., 1999) form. Inside the cell amastigotes multiply by binary fission and when they reach a high density, they transform into trypomastigotes that are released into the blood where they can invade new cells and propagate the infection. The premature lysis of infected host cells can also liberate amastigotes that will contribute to the intra-host propagation of the infection. Other forms of transmission in humans include non-tested blood transfusion, organ transplantation, vertical transmission and oral infection by ingestion of food contaminated by infected triatomine feces (Coura and Borges-Pereira, 2010).

Chagas disease presents two main forms, the acute and the chronic phase. The acute phase lasts between four and eight weeks after parasite inoculation and is asymptomatic in most cases, or could exhibit unspecific symptoms like fever, muscle pain, subcutaneous edema, hepatosplenomegaly and lymphadenopathy (Rassi et al., 2010). In the chronic phase 60-70% of the patients do not develop symptoms or detectable pathology, despite being seropositive for *T. cruzi.* The remaining 30-40% of the patients, after a period of 10 to 30 years, might develop cardiac (Chronic Chagas Cardiomyopathy, CCC), gastrointestinal (megacolon or megaesophagus), neurological pathologies, or mixed forms of the previous manifestations (Fernandes and Andrews, 2012; Coura, 2015; Lewis and Kelly, 2016).

**Figure 1:**
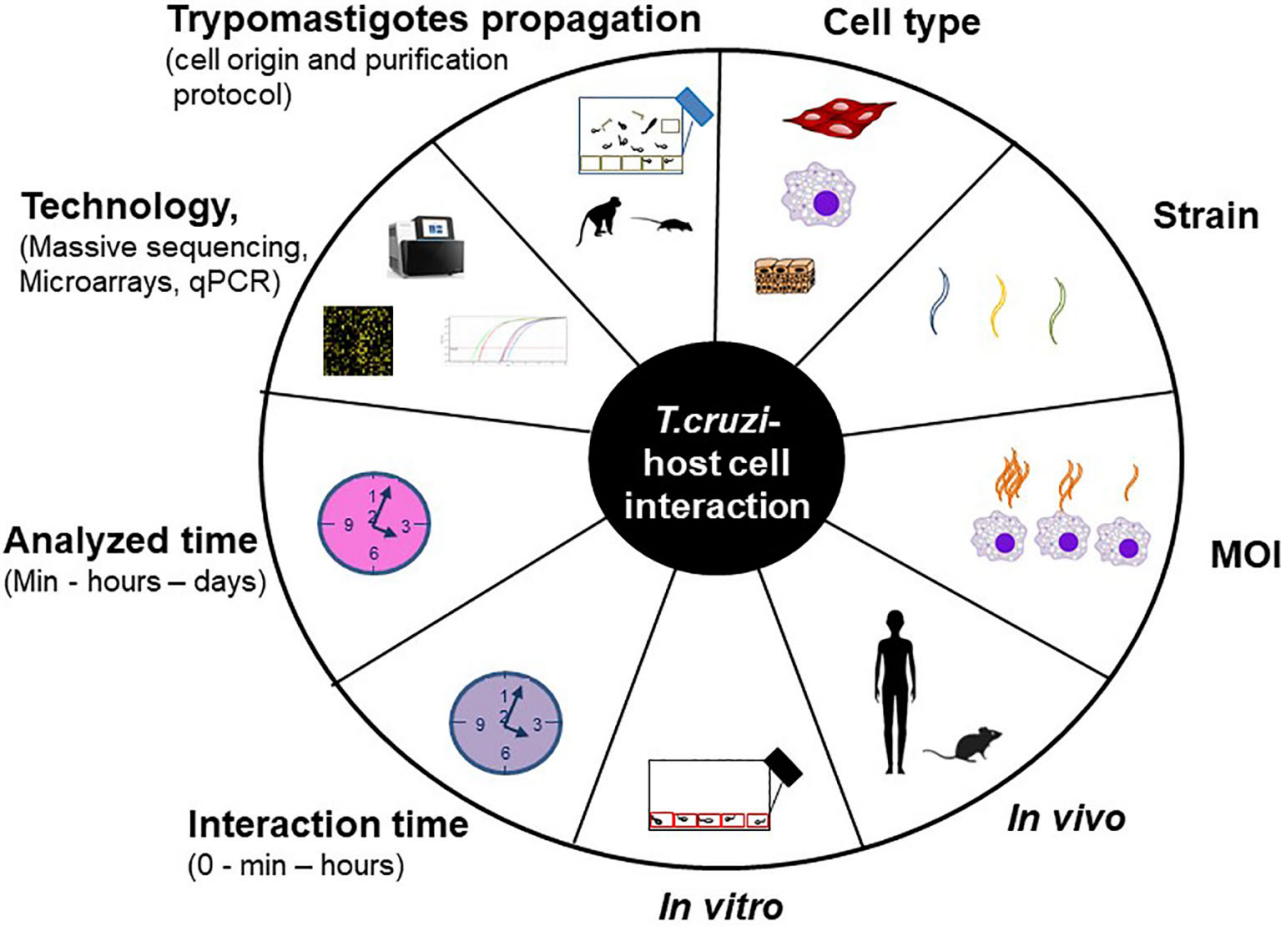
Experimental variables that impact on *T. cruzi* – host cell interaction studies.

The understanding of the host-pathogen interaction process involves the study, among others, of the changes in gene expression provoked by the parasite in the host cell. For this purpose different models are used, both *in vitro* (cell cultures) and *in vivo* (mainly mice) infections. The host cell infection shows considerable heterogeneity at each step of this process, and the specific result may vary with each unique combination of parasite strain, stage (trypomastigotes and/or amastigotes) and host cell type (Epting et al., 2010). Besides, many experimental variables can influence the final result, such as the protocol used to propagate and purify trypomastigotes (Libisch et al., 2020), the interaction time between the parasite and the host cell, the multiplicity of infection (MOI, defined as the ratio of parasites to host cells), the analyzed time, the technology used (qPCR, microarrays or massive sequencing) and the data analysis layout. In addition, it is well known that long lasting *T. cruzi* cultures can suffer a decrease in the infectivity capacity (Contreras et al., 1998). **Figure 1** summarizes the main factors that can vary through the different studies, those which we consider most important are analyzed in this work.

## *T. cruzi* Strain Variability

It has been shown that different *T*. *cruzi* strains exhibit biological differences, such as virulence, infectivity, pathogenicity, among others (Vago et al., 1996; Andrade et al., 1999; Vago et al., 2000; Buscaglia and Di Noia, 2003; Andrade et al., 2010). In an effort to understand this variability, the different strains have been classified into six main groups, known as discrete typing units called DTU (TcI to TcVI). This term was proposed to describe a set of strains that are genetically more related to each other than to any other strain. The first four DTUs are non-hybrid lineages while TcV and TcVI are hybrids of TcII and TcIII (Zingales et al., 2012). However, there exists a high genetic and biological diversity of *T. cruzi* strains even at the intra-DTU level (Berná et al., 2019) and it is known to impact the host response to infection. For example, by comparing the gene expression profiles of two *T*. *cruzi* TcVI strains that display contrasting virulence phenotypes (CL14 and CL Brener which present low and high *in vivo* virulence, respectively), Belew et al. (Belew et al., 2017) found a central role for genes encoding surface proteins associated with the differentiation from intracellular replicative amastigotes to infective trypomastigotes. In order to understand this phenotypic variability, it is important to consider the complex *T. cruzi* genome with its highly repetitive nature: 5–10% of the genome is composed of a 195 bp satellite sequence, and the remaining 40-45% of the repetitive DNA is composed of multi-gene families, tandem repeats and retrotransposable elements (El-Sayed et al., 2005b). The main multi-gene families are: transialidases (TS), mucins (heavily glycosylated proteins), the mucin-associated surface proteins (MASP), GP63, the families DGF-1 (dispersed gene family) and RHS (retrotransposon hotspot) proteins. Our group has found important differences in copy numbers for some of these protein families depending on the DTU (TCC/TcVI and Dm28c/TcI) (Berna et al., 2017; Berna et al., 2018). Wang et al. (2021) also showed important differences between two *T. cruzi* strains (Brazil/TcI and Y/TcII), where all gene families exhibit a broad range of sequence identity between their members, especially TS, MASP, mucins and RHS. In addition, a repeat-driven recombination and generation of antigenic diversity was recently proposed (Talavera-Lopez et al., 2021). These genomic features allow the parasites to display great plasticity and the ability to invade almost any type of nucleated cell. For sure, ongoing works will improve genome assemblies and gene annotations, which in turn will contribute to a better understanding about parasite virulence and plasticity, as well as will allow for a better assessment on the parasite proteins triggering the *T. cruzi-*host interaction.

We also want to highlight that the specific strain does not only impact *T. cruzi* host cell interaction studies, but also on infections with other trypanosomatids. For instance, for another intracellular trypanosomatid as *Leishmania major*, infection by different strains results in different levels of virulence (Markikou-Ouni et al., 2012). In the case of *Trypanosoma brucei*, an extracellular parasite, different strains also show variable virulence (Gitonga et al., 2017).

## The Invasion Process

To succeed in the invasion process the parasites must attach to the host cell surface prior to entry. Most of the interactions between the parasite and the host cell occur through different proteins belonging to multigene families (Woolsey et al., 2003; El-Sayed et al., 2005a; De Pablos et al., 2011). In the case of mucins, they facilitate the adhesion of the trypomastigote to the host cell and its subsequent invasion and also participate in immune response evasion (Andrews and Whitlow, 1989). Transialidases (TS) transfer sialic acid from the host cell membrane to the parasite’s glycoproteins and favors the parasite interaction with the host cell during the invasion process (Woolsey and Burleigh, 2004; Mott et al., 2009). MASP proteins, present in the membrane of trypomastigotes, participate in the invasion of the host cell and in host immune evasion mechanisms (De Pablos et al., 2011; Dos Santos et al., 2012). Individual members of the protease GP63 family also play a role in the infection process (Kulkarni et al., 2009). There are fewer studies about the DGF-1 and RHS families, but members of the former are expressed on the trypomastigote surface and might be associated with the ability of *T. cruzi* to bind extracellular matrix proteins (Kawashita et al., 2009), and the RHS family may play a role in modulating the chromatin structure but also they may participate, possibly as an adjuvant, in the interaction with the mammalian host (Bernardo et al., 2020). After the attachment to the host cell, the parasite enters into the cell. The mechanisms and route of cell invasion vary greatly with the host cell type since *T. cruzi* trypomastigotes are capable of directly invading both professional and non-professional phagocytic cells. In macrophages, trypomastigotes induce their uptake by engaging Toll-like receptors (Maganto-Garcia et al., 2008). In the case of non-professional phagocytic cells, at least two major pathways have been characterized. The first relies upon calcium-mediated signaling at the surface for lysosomal trafficking, dependent upon actin polymerization and microtubules (Schenkman et al., 1991; Tardieux et al., 1994), while the second is a plasma membrane-mediated invagination involving PI3 kinase signaling and independent of actin polymerization (De Souza, 2002; Andrade and Andrews, 2005; Burleigh, 2005). The specific mechanism by which *T. cruzi* invades the host could also depend on the strain (Neira et al., 2002). In any case, the interaction between the parasite and the cell will trigger the transduction of different signals depending on the involved specific host cell receptors and the parasite surface molecules. As it is known, most of these signal transduction pathways will ultimately impact the activity of different transcription factors, resulting in changes in gene expression, which can be considered as a reprogramming of host cells.

Independently of the specific invasion process and the parasite stage (trypomastigotes or amastigotes), components of the host endosomal-lysosomal system such as early endosomes, late endosomes and lysosomes, participate in the formation of a parasitophorous vacuole with an acidic pH (Ley et al., 1990). In the vacuole, trypomastigotes release trans-sialidase, that will remove sialic acid residues from the membrane, making it sensitive to the action of Tc-Tox (a peptide that has homology with the factor 9 of the human complement, Andrews and Whitlow, 1989), which probably contributes to the disintegration of the parasitophorous vacuole (De Souza et al., 2010). The acidic pH favors the conversion of trypomastigotes into amastigotes and the escape to the cytoplasm forto the initiation of amastigotes replication (Ley et al., 1990). Regarding the invasion kinetics (the time the parasite takes to escape from the vacuole and start replication), there are some studies that analyze, with both specific DTUs and host cells, aspects of this process. Ley et al. found that two hours after interaction between human monocytes and parasites from the Y strain (trypomastigotes or amastigotes), 70% of the parasites are in disrupted vacuoles or free in the cytosol (Ley et al., 1990). Stecconi-Silva et al. found that there is a population of trypomastigotes of the G strain that leaves the vacuole at 7 to 8h and another set that leaves the vacuole between 12-15h post-invasion of HeLa and Vero cells (Stecconi-Silva et al., 2003). Dias et al. found that the JG and Col1.7G2 trypomastigotes escape from the vacuole at 8 to 12h post-invasion of primary neonatal cardiomyocytes cultures (Dias et al., 2017). Once free in the cytoplasm the parasites start replication to finally, 3-5 days post infection, lyse the cell.

Although many studies analyze the host cell response to *T. cruzi* infection, the comparison between them becomes very difficult due to significant differences in the incubation periods and analyzed times after infection, which encompass distinct biological events during the infection process. These differences together with the particular molecular interactions established by the specific combination of strain and cell type will impact the host transcriptomic and functional response in very different ways.

## Are Transcriptomic Experiments Comparable to Each Other?

For *in vivo* gene profiling experiments mice are the animal model of choice for the majority of the studies. Despite the study of the immune response using mice models have yielded important insights into the workings of the human immune system, there are also significant differences in both innate and adaptive immunity including the balance of leukocyte subsets, Toll-like receptors, FcR, immunoglobulins subsets, the B and T cell signaling pathway components, cytokines and cytokine receptors, Th1/Th2 differentiation, Ag-presenting function of endothelial cells, among others (Mestas and Hughes, 2004). Regarding general metabolic features between mice and humans, mice have a higher mass-specific metabolic rate and therefore a weak capacity to maintain cellular homeostasis, while humans have a lower mass-specific metabolic rate and a strong capacity to maintain cellular homeostasis (Demetrius, 2004; Demetrius, 2005). All these differences show that there are important caveats to consider that could impact the *in vitro* results between experiments done with mice or human origin cells, and when it comes to extrapolating results from *in vivo* mice models to humans.

To perform transcriptomic host – *T. cruzi* experiments, propagation of parasites in order to perform the infection assays constitutes a variable that is not usually considered. One of the most common systems to do the parasite propagation is the *in vitro* infection of Vero (or other types of monkey-derived) cells (Duran-Rehbein et al., 2014). We recently demonstrated that when parasites are grown in a cell line genetically close to the target cells in the infection experiment, false-positive results may occur in the subsequent transcriptomic analysis (Libisch et al., 2020). This contamination happens because, in the trypomastigote RNA purification process, RNA coming from the cell line used to propagate the parasites is also extracted. This observation showed that particular attention must be paid both in the selection of the cell origin used for parasite propagation (the further away phylogenetically from the target cells, the better) and in the protocol used to purify the trypomastigotes in order to avoid this type of contamination. Another important aspect of the experimental conditions that could impact the final host response is the MOI, which not only can affect the percentage of infected host cells but also could modulate the type of host cell death, as has been observed in some bacterial infections (Lee et al., 2006; Willingham et al., 2007). Finally, the different technologies used to obtain the transcriptomic data (q-PCR, microarrays or massive sequencing) and the specific type of data analysis can impact the final results.

All these variables make it difficult to compare the different results, and not taking them into account can lead to incorrect conclusions. **Table 1** shows different transcriptomic studies performed by diverse groups where they analyze the host cell response to *T. cruzi* infection. We summarize 11 *in vivo* and 18 *in vitro* studies. In the case of the *in vivo* studies six involved mice and five CCC patient samples. The number of parasites used to establish the mice infections were as few as 50 parasites to as many as 10^6^ parasites, and the analyzed time varied between 3dpi to 8 months. In the case of the *in vitro* studies, the MOI varied between 1 to 80, and the experiments were done in cells derived from mice (4), humans (11) and rats (3). The host cell type more frequently analyzed in the *in vitro* studies were fibroblast (6) and cardiomyocytes (4) followed by epithelial HeLa cells (3), rat myoblast (3), human placenta (1) and endothelial cells (1). The cell lines more frequently used to propagate the parasites were cells derived from monkeys (Vero and LCMK2 lines). The interaction time varied between 0 to 60h and the analyzed time between 30min to 96hpi.

**Table 1:** Different *T. cruzi*-host cell interaction studies incorporating transcriptomics of the infected host cell.

N	Strain/DTU	PM	Infection Model	MOI	IT	AT	Methodology	Reference, year
1	Sylvio/TcI	C2C12	Male mouse C3H/HeN (6-8 weeks)	10^6^/mice	NA	3, 37 y 110dpi	Microarrays (Clontech)	(Garg et al., 2003)
2	ND	NA	Heart Tissues from CCC or DCM patients	NA	NA	NA	Microarrays (Affymetrix)	(Cunha-Neto et al., 2005)
3	Tulahuen/TcII	*In vitro* from epis	Murine fibroblast cell line NIH3T3	1:1	0	8dpi (epis at 37°C)	Microarrays (Custom)	(Imai et al., 2005)
4	Brazil/TcI Y/TcII	ND	Male Mice CD-1	10^4^/mice	NA	30, 60, 90, 120, 150, 180dpi	Microarrays (Custom)	(Mukherjee et al., 2008)
5	Tulahuen/TcII	ND	HeLa	10:1	60h	12hpi	Microarrays (Affymetrix)	(Shigihara et al., 2008)
6	Y/TcII	LLCMK2	Fibroblasts, endothelial and smooth muscle human cells	30:1	2h	24hpi	Microarrays (Affymetrix)	(Costales et al., 2009)
7	Brazil/TcI	L6E9	Cardiomyocytes from neonatal mice (C57BL/6)	5:1	24h	48hpi	Microarrays (Custom)	(Goldenberg et al., 2009)
8	Y/TcII	LLCMK2	Skin from site of infection of BALB/c mice	10^6^/mice	NA	24hpi	Microarrays (Affymetrix)	(Chessler et al., 2009)
9	Brazil/TcI Y/TcII CLBr/TcVI Tulahuen/TcII	L6E9	L6E9, rat myoblast	1:1	48h	72hpi	Microarrays (Custom)	(Adesse et al., 2010)
10	Tulahuen/TcII	Rat Heart myoblast	Primary human coronary artery smooth muscle	10:1	0	30, 60, 120, or 180min	Microarrays (Custom)	(Nde et al., 2010)
11	Colombiana/TcI	LLCMK2	C57BL/6 male and female mice	ND	NA	8 month	Microarrays (Custom)	(Soares et al., 2010)
12	Dm28c/TcI	Vero	Primary mouse cardiomyocytes obtained from 18-day-old embryos	10:1	6h	1, 2, 4, 6, 12, 24, and 48hpi	Microarrays (Affymetrix)	(Manque et al., 2011)
13	Tulahuen/TcII	L6E9	Primary endothelial cells (EC) from the epididymal fat from rats	2:1	0	2, 18, 48hpi	Microarrays (Affymetrix)	(Tanowitz et al., 2011)
14	Y/TcII	LLCMK2	6-8 wk C57BL/6 mice	10^3^/mice	NA	18dpi	Microarrays (Affymetrix)	(Silva et al., 2013)
15	Tulahuen/TcII	LLCMK2	HeLa	5:1	2h	18 y 72hpi	Genome wide RNAi.	(Caradonna et al., 2013)
16	Dm28c/TcI	Vero	HeLa	10:1	4h	0, 3, 6hpi	Microarrays (Agilent)	(Chiribao et al., 2014)
17	Tulahuen/TcII	Rat Heart myoblast	Human Primary cardiomyocytes	10:1	0	15, 30, 60, 90 and 120 min	Microarrays (Affymetrix)	(Udoko et al., 2016)
18	Y/TcII	LLCMK2	Human foreskin fibroblasts	ND	2h	4,6,12,24,48 y 72hpi	Sequencing (Illumina)	(Li et al., 2016)
19	Sylvio/TcI Y/TcII	Vero	Human foreskin fibroblasts	10:1	2h	4, 12, 20, 24, 30, 48, 72 and 96hpi	Sequencing (Illumina)	(Houston-Ludlam et al., 2016)
20	CLBr/TcVI CL14/TcVI	LLCMK2	Human foreskin fibroblasts	80:1	2h	60-96hpi	Sequencing (Illumina)	(Belew et al., 2017)
21	NA	NA	Blood samples from CCC patients.	NA	NA	NA	Microarrays (Illumina)	(Ferreira et al., 2017)
22	NA	NA	Heart biopsies from CCC patients.	NA	NA	NA	Microarrays (Agilent)	(Laugier et al., 2017)
23	NA	NA	Human placental RNA samples from CCC patients	NA	NA	NA	Sequencing (Illumina)	(Juiz et al., 2018)
24	Y/TcII	Vero	Explants of human placenta	10^5^-10^6^/ml	0	2 or 24hpi	Microarrays (Agilent)	(Castillo et al., 2018)
25	Dm28c/TcI	Vero	Human Primary cardiomyocytes	10:1	2h	0, 2, 4, 6,12hpi	Microarrays (Agilent)	(Libisch et al., 2018)
26	ND Y/TcII	NA LLCMK2	CCC and DCM patients C57BL/6 6-8 weeks	NA 10^3^/mice	NA NA	NA 18dpi	Microarrays (Agilent)	(Silva et al., 2018)
27	Col1.7G2/TcIJG/TcII	SWISS mice	6-8-week-old male BALB/c mice	50	NA	15dpi	Sequencing (Illumina)	(De Castro et al., 2020)
28	Brazil/TcI Y/TcII CLBr/TcVI Tulahuen/TcII	L6E9	L6E9	1:1	48h	72hpi	Microarrays (Custom)	(Nisimura et al., 2020)
29	CLBr/TcVI CL14/TcVI	LLCMK2	Human foreskin fibroblasts	80:1	2h	60 and 96hpi for infected samples. 48 and 60hpi for controls	Sequencing (Illumina)	(Oliveira et al., 2020)

PM, Parasite Propagation Model; MOI, Multiplicity of Infection; IT, Interaction Time; AT, Analyzed Time; NA, Not Applicable; ND, No Data available; C2C12, mice myoblast cells; LLCMK2, Kidney cells from Macaca mulatta; Vero, Kidney cells from Cercopithecus aethiops; L6E9, myoblast Rat cells.

All the variables mentioned above also affect transcriptional changes induced by other intracellular trypanosomes, as *Leishmania* (different infection models, the source and origin of the macrophages, parasite species, levels of infectivity, MOI, the used technology, data analysis, and interaction and analyzed times of infection) (Ashwin et al., 2018; Stamper et al., 2019) and probably they will also affect the transcriptional host changes caused by any intracellular microorganism.

## How Is Host Cellular Respiration Affected by *T. cruzi?*

To exemplify the differences between the host responses to *T. cruzi* infection given the experiment design, we will focus the analysis on the respiratory chain and oxidative phosphorylation (OXPHOS) responses **(** **Table 2** **).** First, when comparing *in vitro-*based transcriptomic responses of human primary cardiomyocytes at early times post infection we found, using the Dm28c strain (TcI), upregulation of pathways related to energy metabolism (Libisch et al., 2018), while Udoko et al. (2016) using the Tulahuen strain (TcVI) did not found these changes. Concerning *in vitro* transcriptomic experiments performed in murine (instead of human) primary cardiomyocytes at early time post-infection, Manque et al. (Manque et al., 2011), using the Dm28c strain did not found significant changes in pathways related to energy metabolism, while Goldenberg et al. with the Brazil strain (TcI) observed a down-regulation of the electron transport activity GO term (Goldenberg et al., 2009), although in this last case the analysis was on late time post infection. These comparisons exemplify how transcriptomic *in vitro* results depend on the specific strain, the origin of the infected cells (human or mouse), as well as on the experimental interaction and analyzed times. It is important to highlight that in only one of these studies a functional validation of the OXPHOS response was performed (see below).

**Table 2:** Transcriptomic and functional results from different studies related to the host cell respiration response to *T. cruzi* infection.

N	Strain/DTU	PM	Infection model	MOI	IT	AT	Methodology	Effects on cellular respiration (reference, year)
***In vitro transcriptomic experiments***								
1 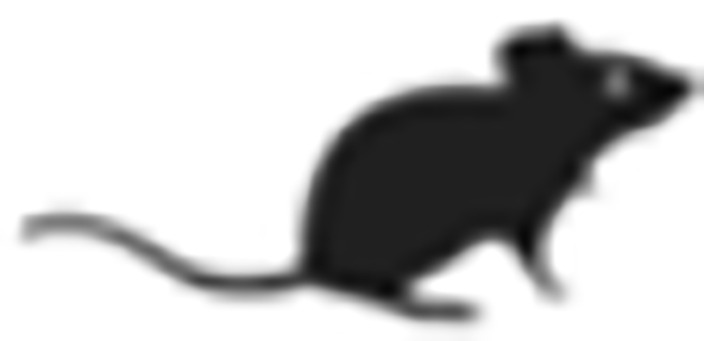	Brazil/TcI	L6E9	Cardiomyocytes from neonatal mice (C57BL/6)	5:1	24h	48hpi	Microarrays (Custom)	Repression of some OXPHOS related genes (Goldenberg et al., 2009)
2 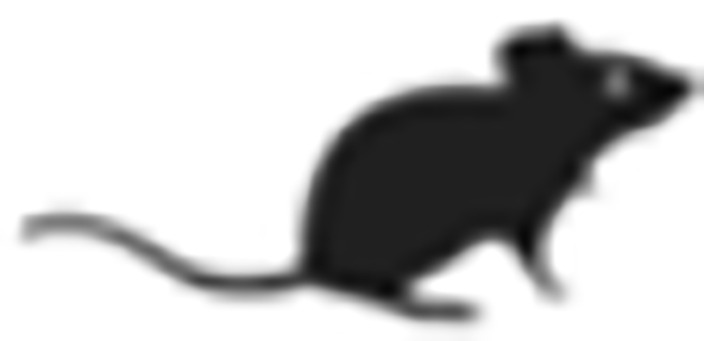	Dm28c/TcI	Vero	Primary mouse cardiomyocytes from embryos.	10:1	6h	1 to 48hpi	Microarrays (Affymetrix)	No significant changes of some OXPHOS (Manque et al., 2011)
3 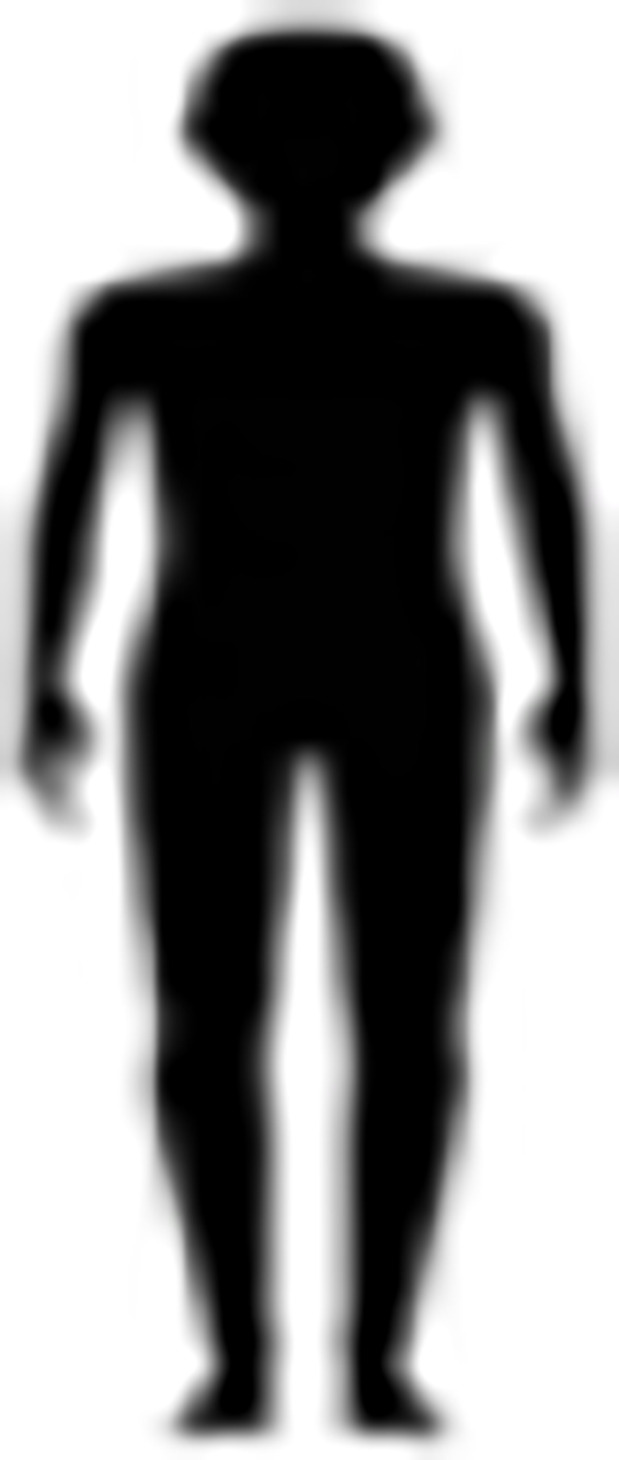	Tulahuen/TcII	Rat Heart myoblast	Primary human cardiomyocytes (PromoCell)	10:1	0	15min to 2hpi	Microarrays (Affymetrix)	No significant differences in pathways related to OXPHOS (Udoko et al., 2016)
4 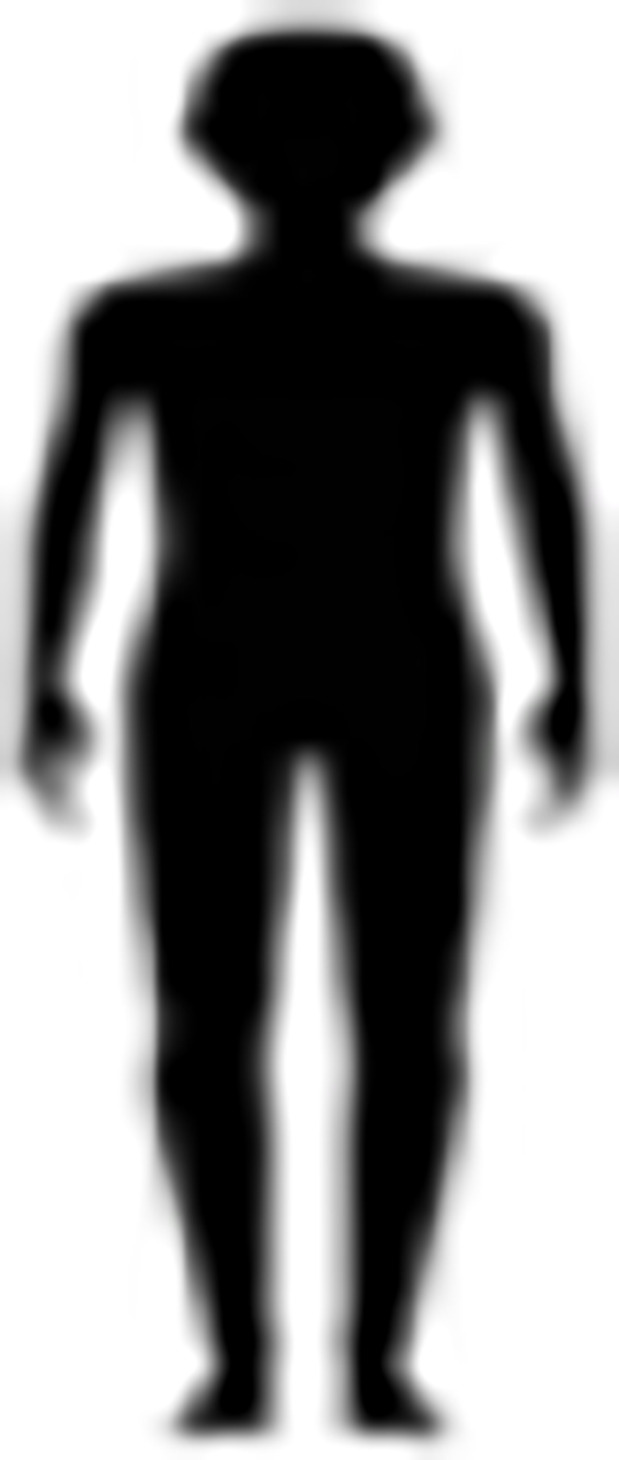	Dm28c/TcI	Vero	Primary human cardiomyocytes (CelProgen)	10:1	2h	0, 3, 6, 12hpi	Microarrays (Agilent)	Up-regulation of OXPHOS related genes (Libisch et al., 2018)
***In vivo transcriptomic experiments***								
5 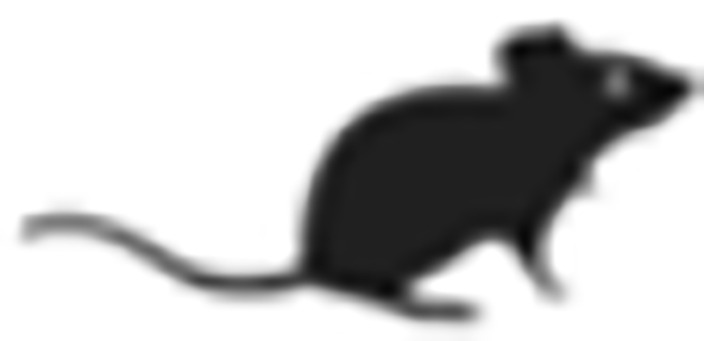	Sylvio/TcI	C2C12	Male mice hearts(C3H/HeN)	10^6/^mice	NA	3, 37, 110dpi	Microarrays (Clontech)	Down-regulation of OXPHOS related genes in cardiac tissue (Garg et al., 2003)
6 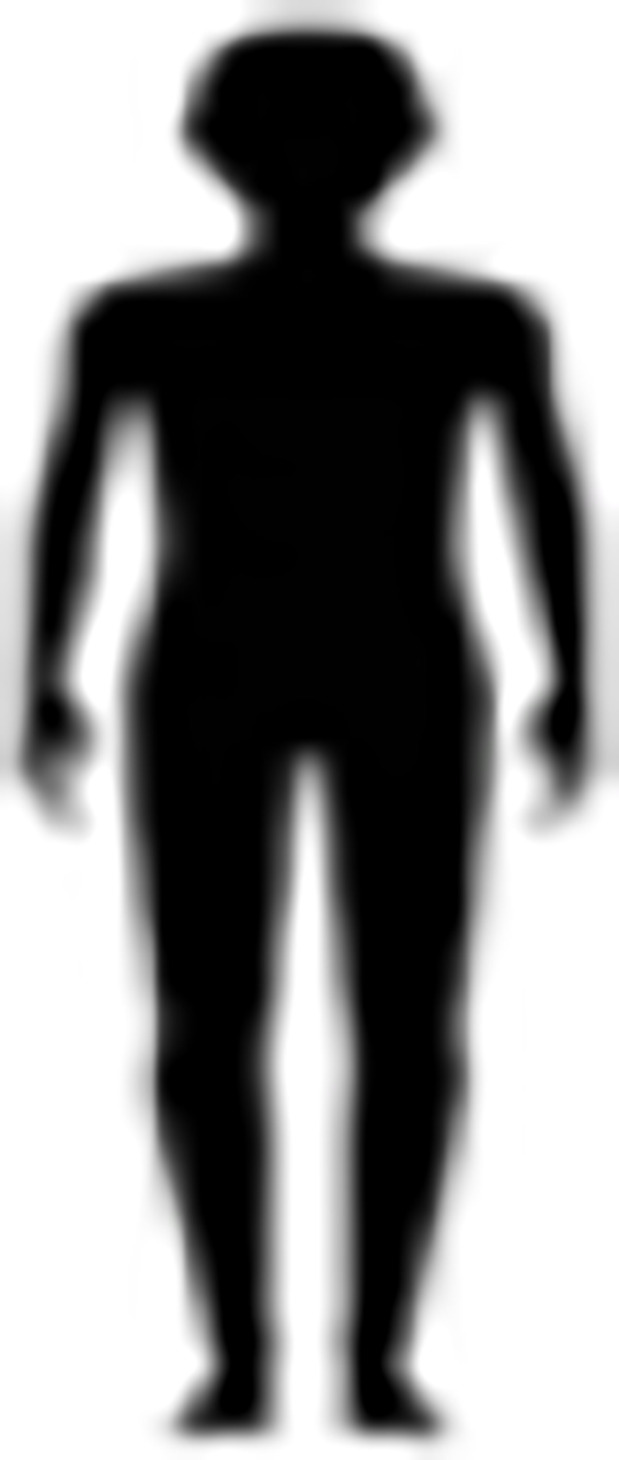	NA	NA	Myocardial tissues from CCC or DCM patients	NA	NA	NA	Microarrays (Affymetrix)	Up-regulation of OXPHOS related genes in CCC patients (Cunha-Neto et al., 2005)
7 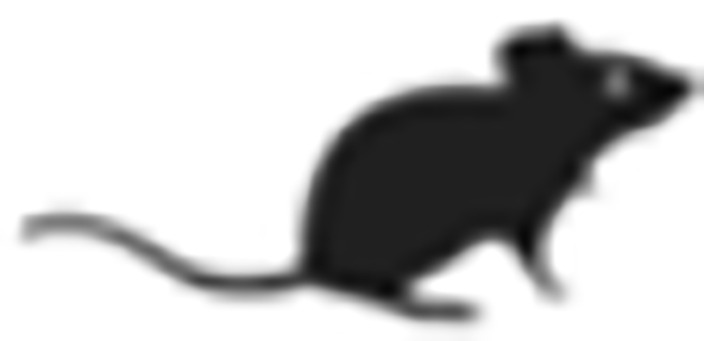	Brazil/TcI Y/TcII	ND	Male mice heart (CD-1)	10^4^/mice	NA	30 to 180dpi	Microarrays (Custom)	Repression of some OXPHOS related genes at the chronic stage (Mukherjee et al., 2008)
8 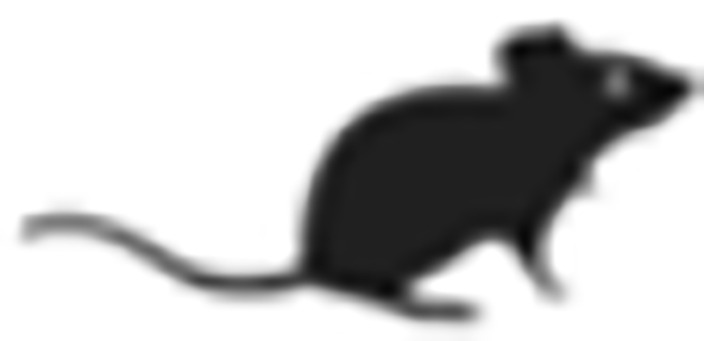	Col1.7G2/ TcI JG/TcII	SWISS mice	Male mice heart (BALB/c)	50/Mice	NA	15dpi	Sequencing (Illumina)	Down-regulation of OXPHOS related genes when using the JG strain (De Castro et al., 2020)
***In vitro functional experiments***								
9 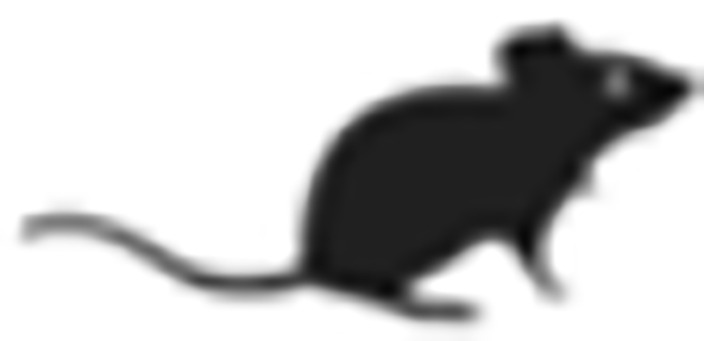	Sylvio/TcI	C2C12	Cardiomyocytes (HL-1 and primary rat cardiomyocytes)	5:1	3h	48hpi	Histochemical staining	Decrease activities of complex I and III in HL-1 infected cardiomyocytes (Gupta et al., 2009)
10 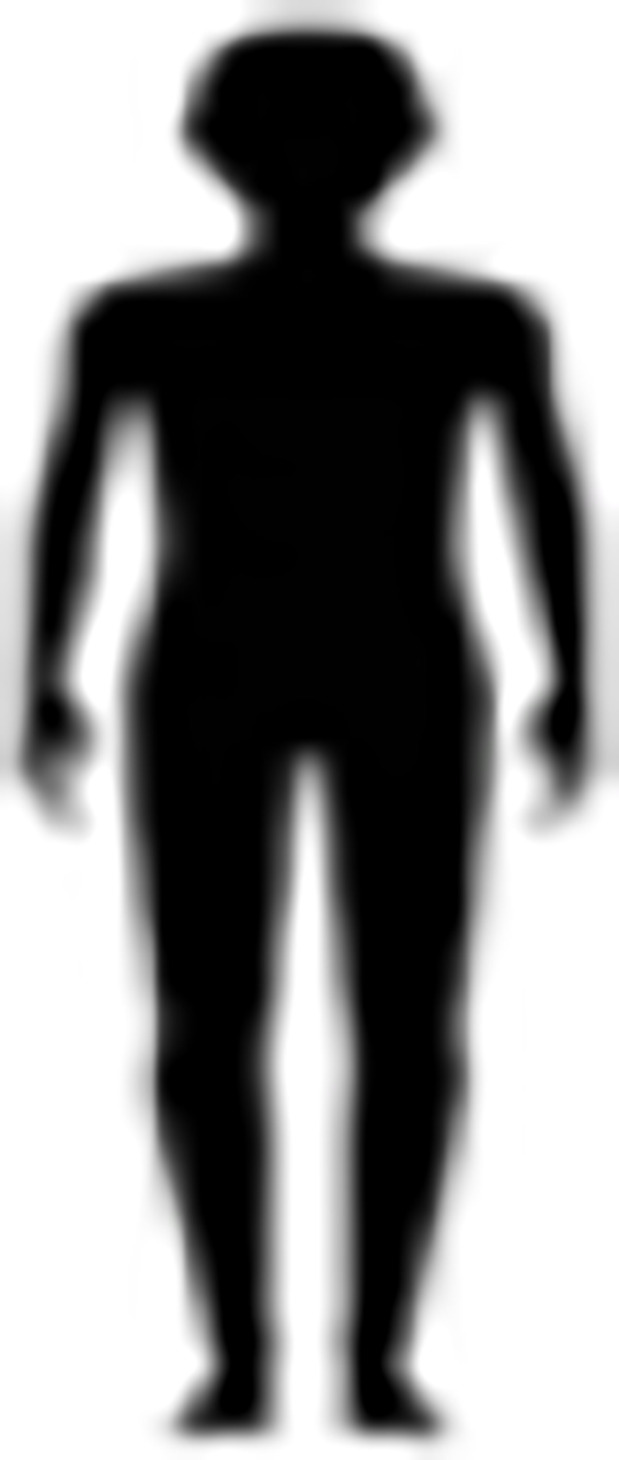	Tulahuen/TcII Y/TcII	LLCMK2	Normal human dermal fibroblasts	50:1	1h	48hpi	Seahorse	OCR increase in infected human fibroblast (Shah-Simpson et al., 2017)
11 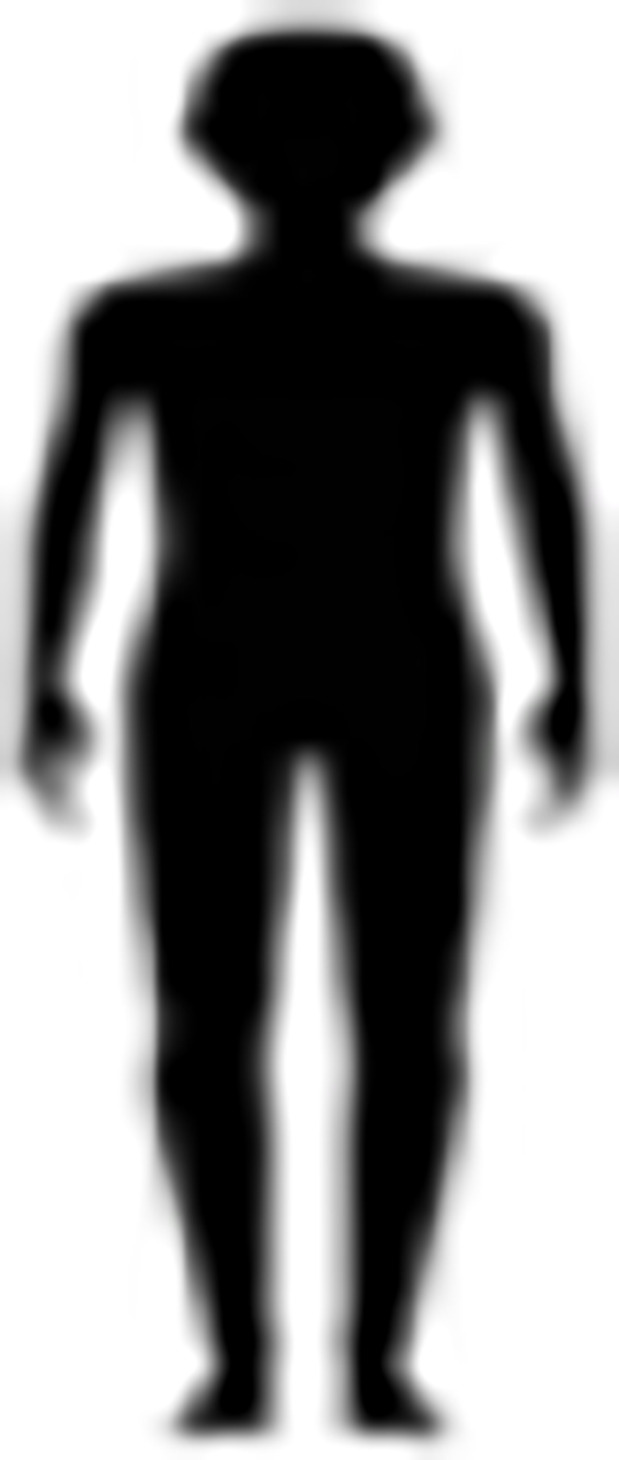	Sylvio/TcI	C2C12	Human THP-1 macrophages	3:1	0	3 and 18hpi	Seahorse	OCR increase in infected human Macrophages (Koo et al., 2016)
12 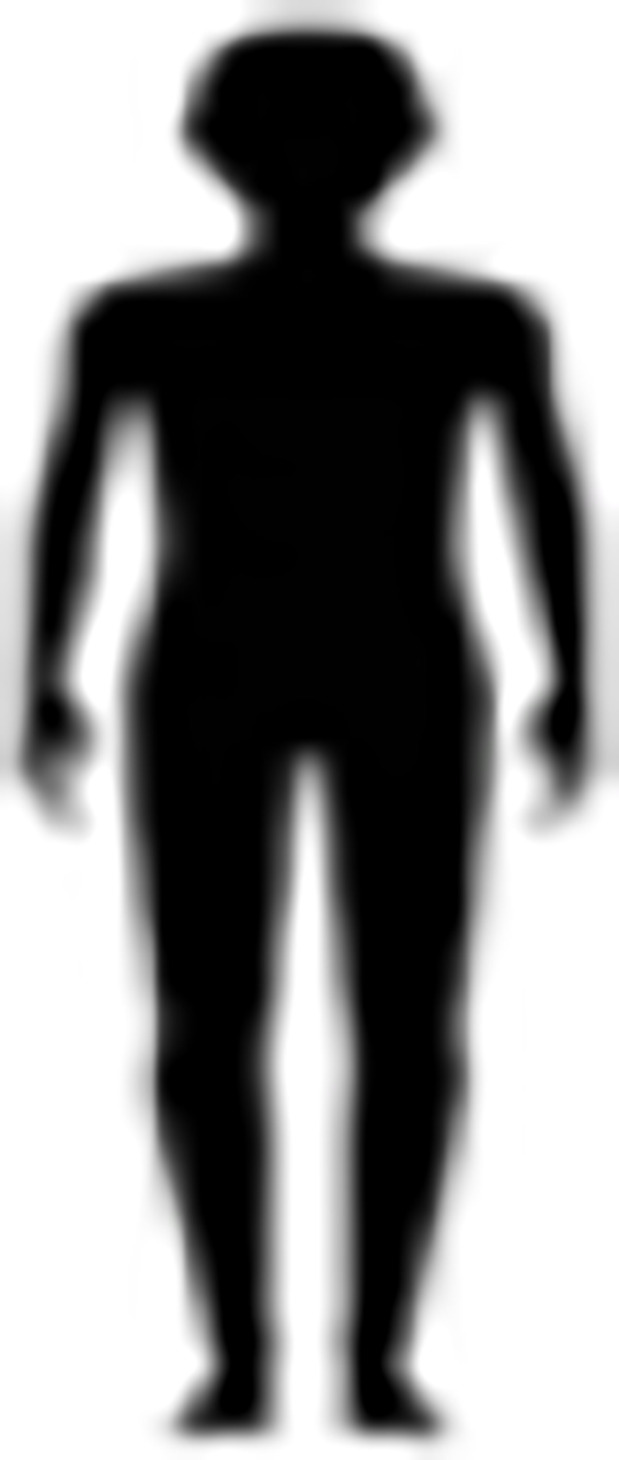	Dm28c/TcI	Vero	Primary human cardiomyocytes (CelProgen)	10:1	2h	6, 17, 24hhpi	Seahorse	Up-regulation of OXPHOS related genes and OCR increase (Libisch et al., 2018)
14 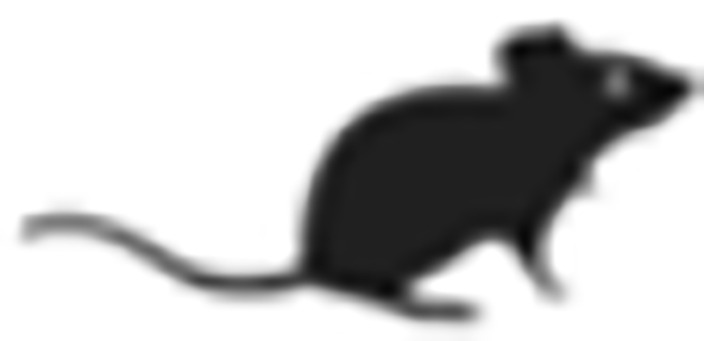	CLBr/TcVI	Vero	Primary mouse cardiomyocytes (BALB/c)	5:1	0	24hpi	Seahorse	OCR decrease in infected mouse cardiomyocytes (Estrada et al., 2018)
***In vivo functional experiments***								
15 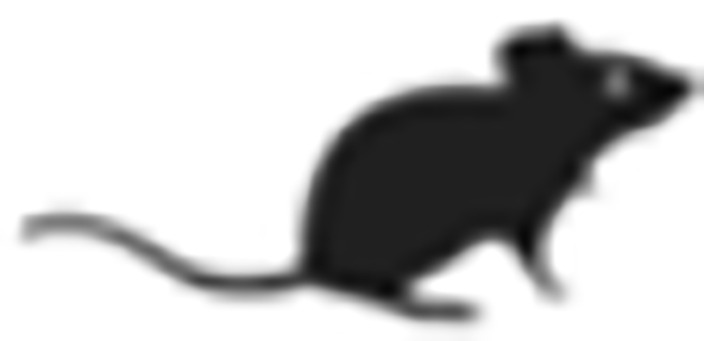	Sylvio/TcI	C2C12	Cardiac mitochondria from male mice (C3H/HeN)	10^4^/mice	NA	3-10dpi 14-40dpi >110dpi	Histochemical staining	Inhibition of the respiratory chain complexes (CI-CV)I n the myocardium of infected mice (Vyatkina et al., 2004)
16 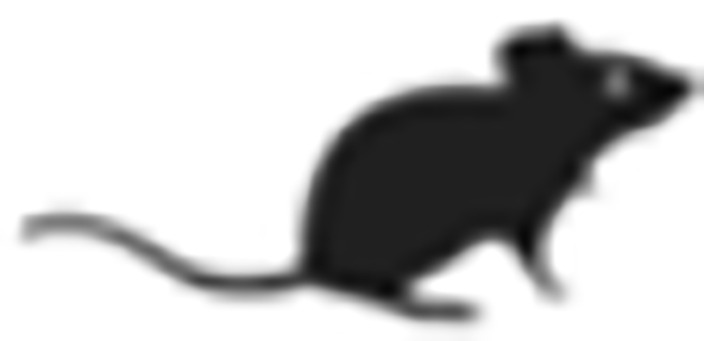	Sylvio/TcI	NA	6-8-week-old male C3H/HeN mice. (heart, stomach, skeletal muscle, colon)	10^4^/mice	NA	20-35dpi 158-180dpi	Measure of antioxidant/oxidant status and mitochondrial function	Oxidative damage and mitochondria decay in acute infection in al tissues, and in heart and stomach in chronic infection, (Wen et al., 2008)
17 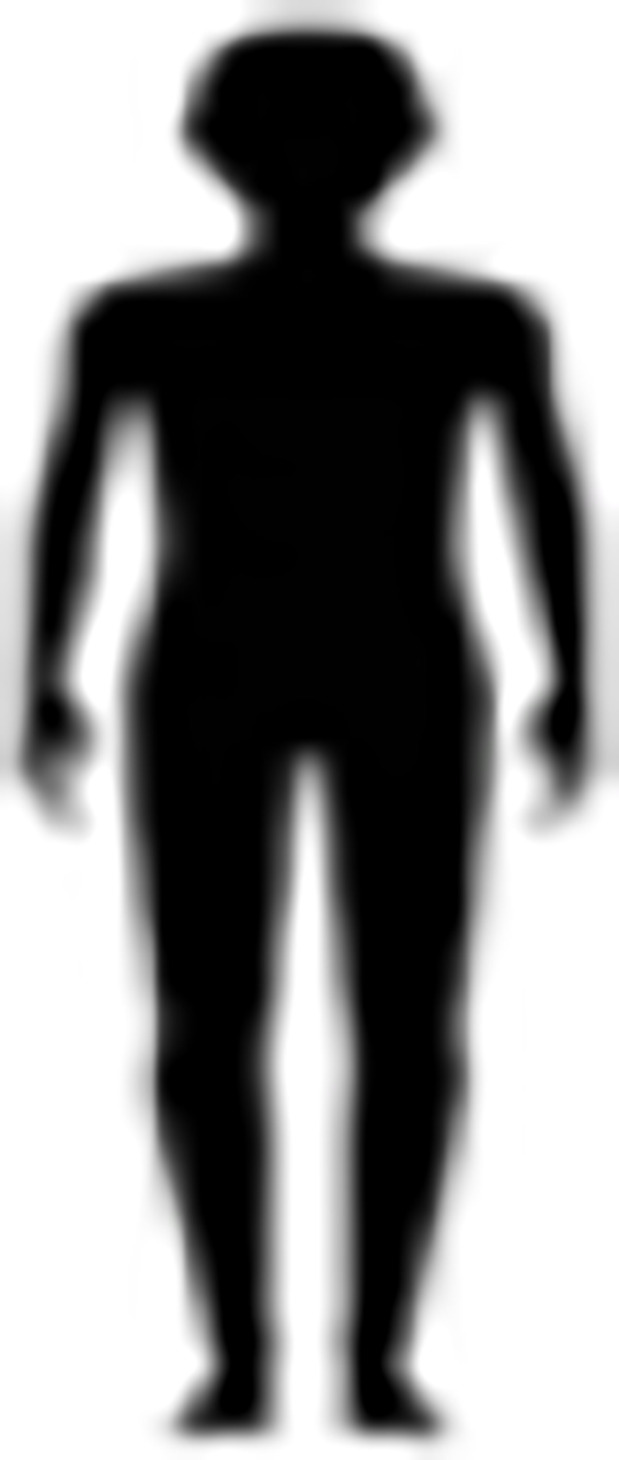	ND	NA	Myocardium homogenates from CCC patients	NA	NA	NA	Western blot	Decrease in components of the creatine kinase system and ATP synthase complex from CCC patients (Teixeira et al., 2011)
18 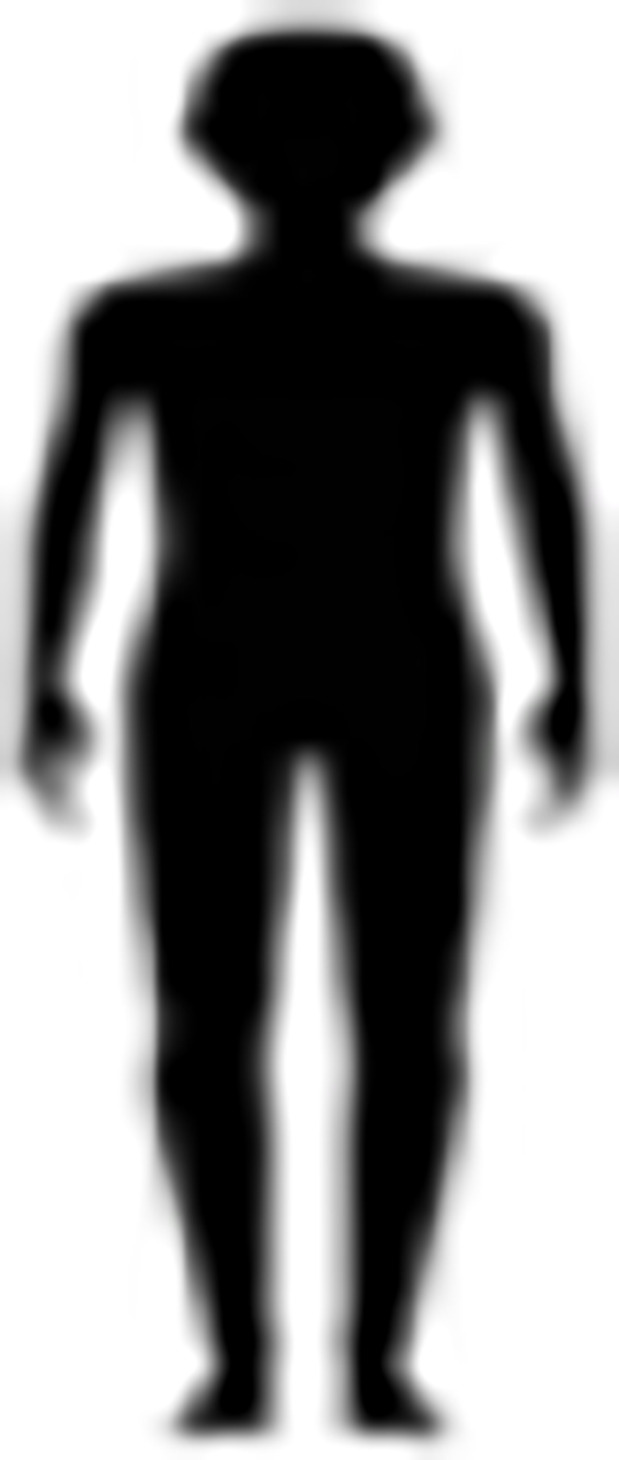	ND	NA	Cardiac biopsies from CCC patients	NA	NA	NA	Western blot and immunohistochemistry	Decrease in protein levels of subunits of the respiratory complexes (CI and CIII) in chagasic hearts biopses (Wan et al., 2012)

The blue and orange color show whether the study found a decrease or an increase in transcriptomic (light color) and functional (dark color) respiration in T. cruzi infected cells, respectively. (PM, Parasite Propagation Model; MOI, Multiplicity of Infection; IT, interaction time; AT, Analyzed time; NA, Not Applicable; ND, No Data available, OXPHOS, Mitochondrial oxidative phosphorylation system; C2C12, mice myoblast cells; LLCMK2, Kidney cells from Macaca mulatta; Vero, Kidney cells from Cercopithecus aethiops; Seahorse, Seahorse extracellular flux analyzer).

By comparing *in vivo-*based transcriptomics data from mice heart infections, Garg et al. (2003) (3-110dpi) using the Sylvio strain (TcI), and Mukherjee et al. (2008) (3-180dpi) using the Brazil (TcI) and Y (TcII) strains, showed a decrease in the energy metabolism pathway. De Castro et al. (2020) analyzed the transcriptomic response of murine hearts infected with the Col1.7G2 (TcI), and JG (TcII) strains, 15dpi, and also found a decrease in pathways related to energy metabolism. Concerning samples from patients with Chronic Chagas Cardiomyopathy (CCC), the study from Cunha-Neto et al. (2005) showed the contrary, an increase in genes related to energy metabolism. These works showed that *in vivo* transcriptomic results could also depend on the origin of the infected cells (human or mouse). Of note, additional specific factors will also impact results, such as the genetic background and the immune status of each cell donor, which should be at least described.

It is important to note that transcriptional changes in response to infection do not imply a correlation with protein levels and/or activity, and hence to the functional response. Regulatory processes occurring after mRNA transcription such as the stability or differential degradation of the RNAs, post-transcriptional modifications, the accessibility to the translation machinery, translational and protein degradation regulation are important factors in controlling steady-state protein levels and specially, post-translational modifications are important factors in controlling protein activity (Vogel and Marcotte, 2012).

Taking these aspects into consideration we looked for studies that had evaluated not only changes at the transcriptional level but also the functional response related to cellular respiration in host cells infected by *T. cruzi*. Looking at functional data related to cellular respiration in mouse and human *in vitro* infection systems, an increase in respiration of different infected human origin host cells was observed: we found that human primary cardiomyocytes infected with Dm28c (17-24hpi) increase respiration and mitochondrial biogenesis (Libisch et al., 2018). Shah-Simpson et al. also found both an increase in respiration and mitochondrial biogenesis of *T. cruzi* infected human dermal fibroblast (48hpi) (Shah-Simpson et al., 2017), which is maintained even when the respiration of *T. cruzi* is specifically inhibited. An increase in respiration (3-18hpi) was also described in human macrophages infected with Sylvio (TcI) by Koo et al. (Koo et al., 2016). In all three cases the respiration was evaluated by the quantification of the oxygen consumption rate (OCR), using the same methodology. On the contrary, Estrada et al. (Estrada et al., 2018) found a decrease in respiration in infected murine cardiomyocytes with the CLBr (TcVI) strain at similar analyzed times (24hpi). Gupta et al. (2009) also described a decrease in the respiration complexes I and III of HL1 mice cardiomyocytes infected with Sylvio (TcI) at 48hpi. These functional works showed again that results are closely related to the origin of infected cells (human or mouse). Regarding *in vivo* infections, Vyatkina et al. (2004) evaluated protein expression of different respiration complexes in cardiac mitochondria from infected mice with the Sylvio strain, finding a decreased expression in some of the complexes (3 to 110dpi). Wen et al. (2008) also found a decreased mitochondrial function in hearts, stomach, skeletal muscle and colon cells of acute mice infection, and also in hearts and stomach of chronic infections. Finally, there are very few studies that analyze the cellular respiration in cells or tissues derived from patients. For example, in a discrete group of CCC heart samples (five patients), Teixeira et al. (Teixeira et al., 2011) found a discrete decrease in the expression levels of the alpha subunit of the ATP synthase enzyme (complex V). Similarly, the work of Wan et al. (2012) in a group of eight CCC heart patients samples, showed a decrease in the Cyt B mitochondrial respiration protein (complex III) and ND1 protein (complex I). Given the diversity of cells present in the heart (cardiomyocytes, fibroblasts, endothelial cells), and in the case of the CCC additional infiltrates of macrophages and T cells, it would be of great importance to perform more studies in a large number of heart biopsies of CCC patients, as well as in cardiomyocytes purified from them.

As mentioned before, working with human or mouse models can have a great impact on the observed host response to infection. Finding an increase in the respiration of infected human cells but a decrease in the respiration in infected cells with a murine origin may be linked to the fact that mice have a basal metabolic rate per gram of body weight approximately seven times higher than that of a human. As a consequence, mice have a lower ability to maintain adequate cellular homeostasis against different types of stress such as reactive oxygen species (ROS) (Demetrius, 2004; Demetrius, 2005). During normal cellular respiration, ROS are usually generated (Scheibye-Knudsen et al., 2015), and particularly they increase in *T. cruzi* infected cardiomyocytes (Gupta et al., 2009; Libisch et al., 2018), and macrophages (Cardoni et al., 1997; Alvarez et al., 2011), and perhaps it is one of the reasons why cells of murine or human origin do not behave the same against infection, at least concerning energy metabolism. Besides there exists an established relationship between the immune system and mitochondrial bioenergetics (Breda et al., 2019), so it is possible that the differences in immune systems between mice and humans also impact on the mitochondrial respiration.

## Does *T. cruzi* Induce Any Common Pathway Independently From Experimental Conditions?

With so many studies performed under so many different experimental variables, we found that activation of the PI3K/AKT pathway is a common response to *T. cruzi* infection. This pathway is an intracellular transduction signal that promotes proliferation, metabolism, cell survival, growth and angiogenesis in response to extracellular signals. Its key components are receptor tyrosine kinases (RTKs), phosphatidylinositol 3-kinase (PI3K), phosphatidylinositol-4,5-bisphosphate (PIP2), phosphatidylinositol-3,4,5-trisphosphate (PIP3) and the serine/threonine protein kinase B/AKT. There are three classes of PI3K. The substrates for class I enzymes are PIP2, which are predominantly found on the plasma membrane where they are phosphorylated to PIP3. The class I enzymes are further divided into two subclasses that differ in their structure and in the stimuli that activate them. The Class Ia enzymes (PI3K α, β, δ) are activated by tyrosine kinase receptors. The class Ib (PI3K γ) is activated primarily by G-protein receptors (Kima, 2016). In all the cases the PI3K class I phosphorylation provokes the AKT phosphorylation and activation. Results obtained by our laboratory in human primary cardiomyocytes and HeLa cells infected with the Dm28c strain show that the PI3K/AKT pathway is activated (Chiribao et al., 2014; Libisch et al., 2018). Wilkowsky et al. and Todorov et al. also found this pathway activated in human and mouse macrophages infected with a TcVI and a TcII strain, respectively (Todorov et al., 2000; Wilkowsky et al., 2001). Caradonna et al. found the PI3K/AKT pathway activated in infected human epithelial (HeLa) cells with the Tulahuen (TcII) strain and its activation was critical for parasite replication (Caradonna et al., 2013). Silva et al. found its activation in mouse cardiomyocytes infected with the Y (TcII) strain (Silva et al., 2018). Chuenkova et al. showed that infected human glial cells of the Schwann cell type with the Sylvio-TcI strain increase the PI3K pathway (Chuenkova and Pereiraperrin, 2009). Nagajyothi et al. also described the PI3K/AKT activation in murine adipocytes infected with the Tulahuen strain-TcII (Nagajyothi et al., 2008), as well as Wilkowsky et al. in murine fibroblasts infected with a TcVI strain (Wilkowsky et al., 2001). PI3K/AKT activation was also demonstrated in patients with CCC by Silva et al. and they also showed that the activation of this pathway in myeloid cells (macrophages) would restrict infection in the heart by protecting cardiomyocytes (Silva et al., 2018). In general, the role of the PI3K/AKT activation is related to its antiapoptotic capacity (Zhou et al., 2000; Yamaguchi and Wang, 2001) and its role in the survival and repair of different cell types has been associated with the ability of the parasite to persist in the host (Nagajyothi et al., 2012). The PI3K pathway could also be necessary for the *T. cruzi* invasion process of non-phagocytic cells as previously mentioned (Woolsey et al., 2003; Burleigh, 2005). In **Table 3** we summarize the high number of works in which, independently of the *T. cruzi* strain, interaction and analyzed time, cell type and cell origin (human or mouse), activation of the PI3K pathway was found. This fact gives strong evidence of the crucial role of this pathway in the interaction of the host cell with the parasite. Chuenkova et al. showed that *T. cruzi* TSs are responsible for activating this pathway in infected Schwann cells (Chuenkova et al., 2001). De Melo et al. showed that a *T. cruzi* trans-sialidase is able to interact with the membrane receptor tyrosine kinase TrKA by promoting invasion of mammalian cells (neuronal, epithelial, and phagocytic cells) and at the same time allowing activation of the PI3K/AKT pathway (De Melo-Jorge and Pereiraperrin, 2007). It is important to consider that activation of PI3K/AKT was reported in infections produced by many different microorganisms. Diehl et al. reviewed different viral strategies which exploit the PI3K/AKT signaling pathway for effective viral replication (Diehl and Schaal, 2013). The activation of this pathway showed to be essential for *Staphylococcus aureus* internalization (Oviedo-Boyso et al., 2011) and also for *Pseudomonas aeruginosa* strain *PAK* internalization (Kierbel et al., 2005). It also played an important role in host defense for some bacterial infections (Yilmaz et al., 2004; Brown et al., 2011; Cremer et al., 2011). Finally, the PI3K signaling plays a role in the host response to *Leishmania* infections (Kima, 2016). Although this pathway appears to be essential in the host response to many types of infections, the particular molecules that activate this pathway are in most cases still unknown. In the case of *T. cruzi*, only TSs in trypomastigotes have been shown to activate this pathway in some mammalian cells (neuronal, epithelial, and phagocytic cells) through TrKA (De Melo-Jorge and Pereiraperrin, 2007). However, it remains to be elucidated which particular trans-sialidases and which particular enzyme of the class Ia PI3K family are involved. It has been shown that *T. cruzi* also activates the PI3Kγ, class Ib enzyme, in macrophages and hearts from CCC patients (Silva et al., 2018). Which particular molecules of *T. cruzi* and G-protein receptors are involved in this activation remains elusive. Understanding the specific molecules and host receptors that participate in the PI3K/AKT activation in different infection models will shed light on its modulation and could be a strategy to control *T. cruzi* infection, replication and/or appearance of clinical signs. Besides, the role of the PI3K/AKT pathway has been extensively studied in the context of cancer, and different drugs targeting PI3K have been developed and employed in clinical trials evaluations (Yang et al., 2019), bringing the option of considering them as repurposed drugs for host-directed therapies in Chagas and others infectious diseases.

**Table 3:** Studies related to the host AKT/PI3K response to *T. cruzi* infection.

Strain/DTU	PM	Infection model	MOI	IT	AT	Methodology	Effects on AKT/PI3K (reference, year)
Y/TcIII 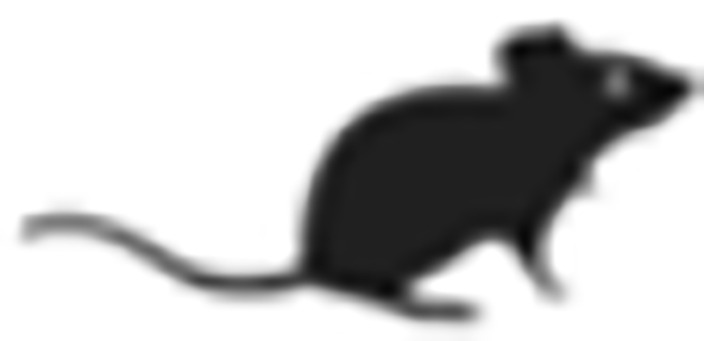	Blood of swiss mice	Swiss Mouse peritoneal macrophages	10:1	0	30min	Phosphoinositide determination and assay of PI3K activity	*T. cruzi* activates PI3K kinases (Todorov et al., 2000)
RA/TcVI 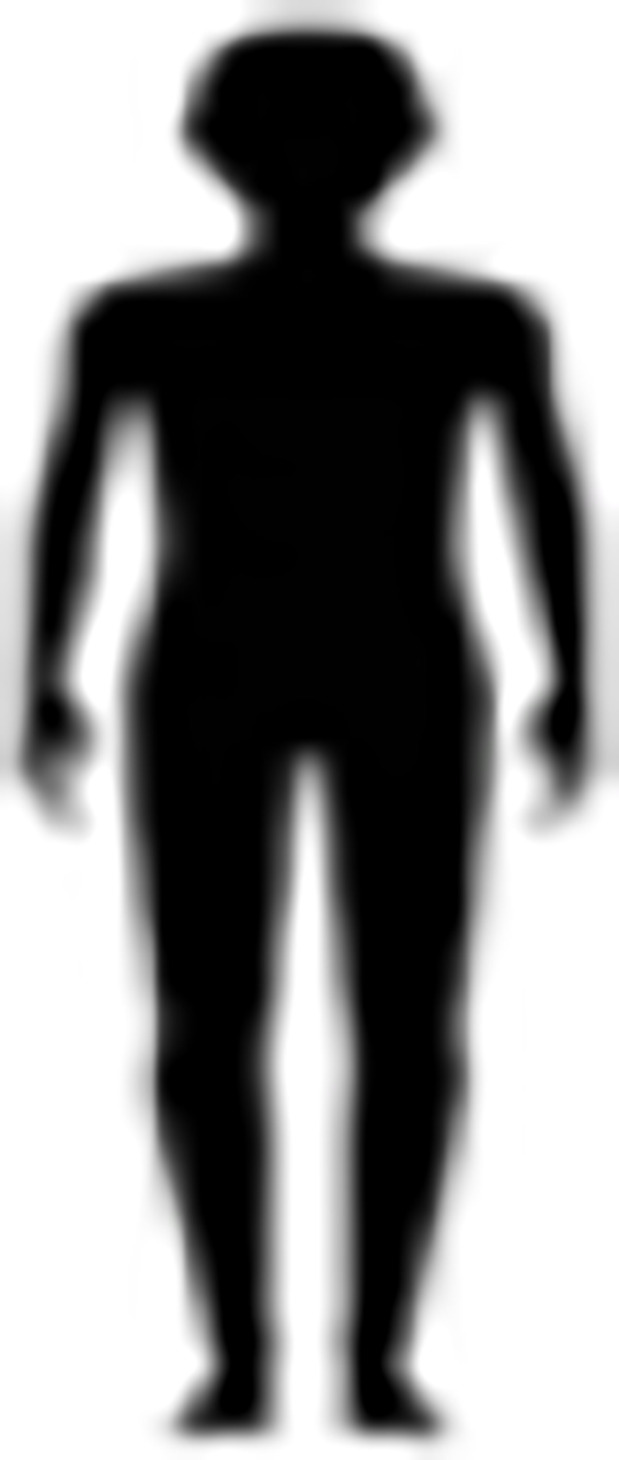	Vero	Human and murine macrophages, NIH3T3 fibroblasts, L6E9	10:1	2h	24hpi	Measure of the percentage of infection with PI3K inhibitors	*T. cruzi* invasion was inhibited in all the analyzed cell types by specific PI3K inhibitors (Wilkowsky et al., 2001)
Tulahuen/TcII 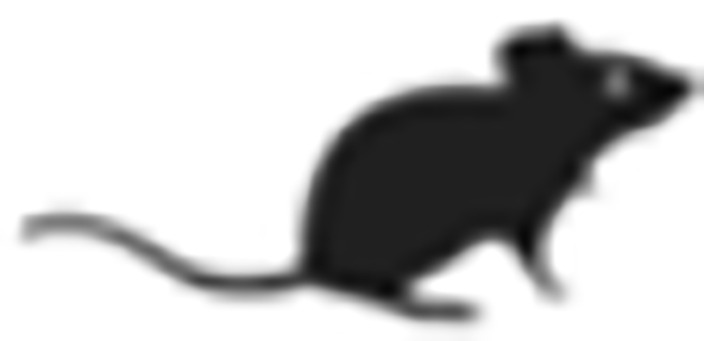	A/J mice and L_6_E_9_	Adipocytes from 3T3-L1 murine fibroblasts	2:1 to 5:1	0	96hpi	Immunoblot	Increase expression of PI3K and AKT activation in infected adipocytes (Nagajyothi et al., 2008)
Sylvio/TcI 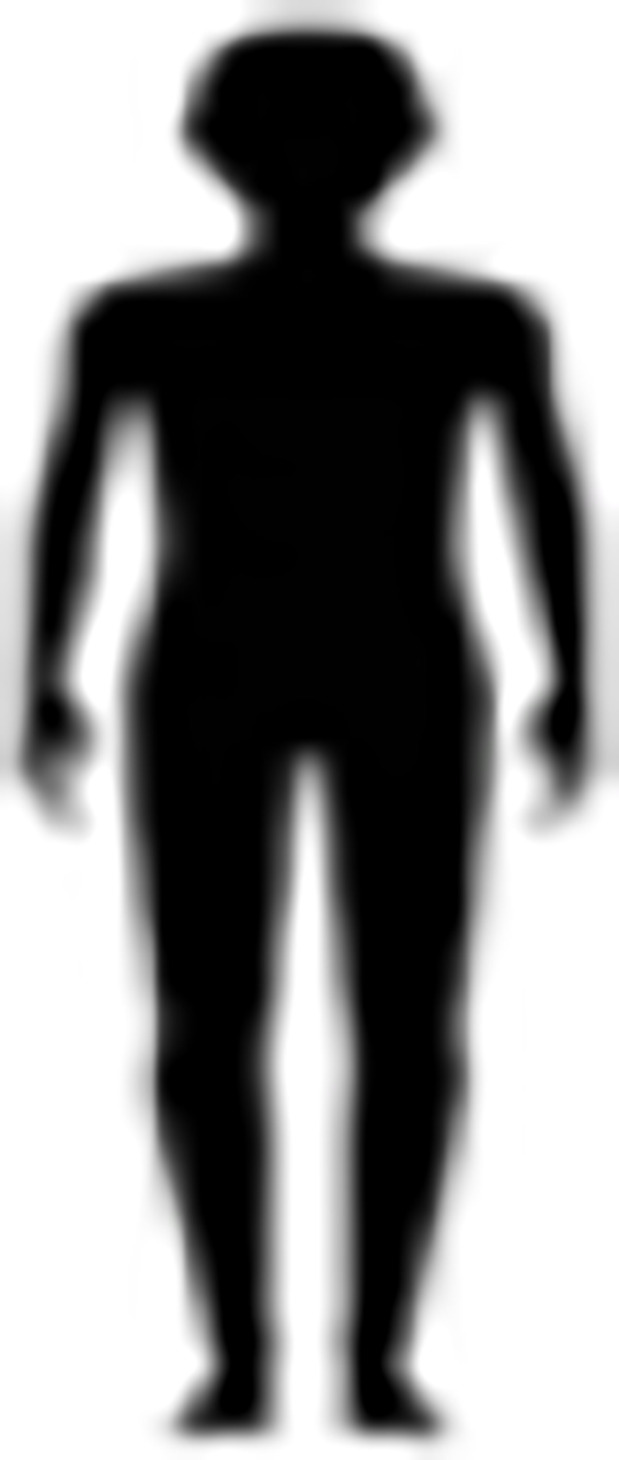	ND	Human Schwann cells	50:1	2-3h	3-5dpi	Microarrays (Custom)	*T. cruzi* targets AKT (Chuenkova and Pereiraperrin, 2009)
Tulahuen/TcII 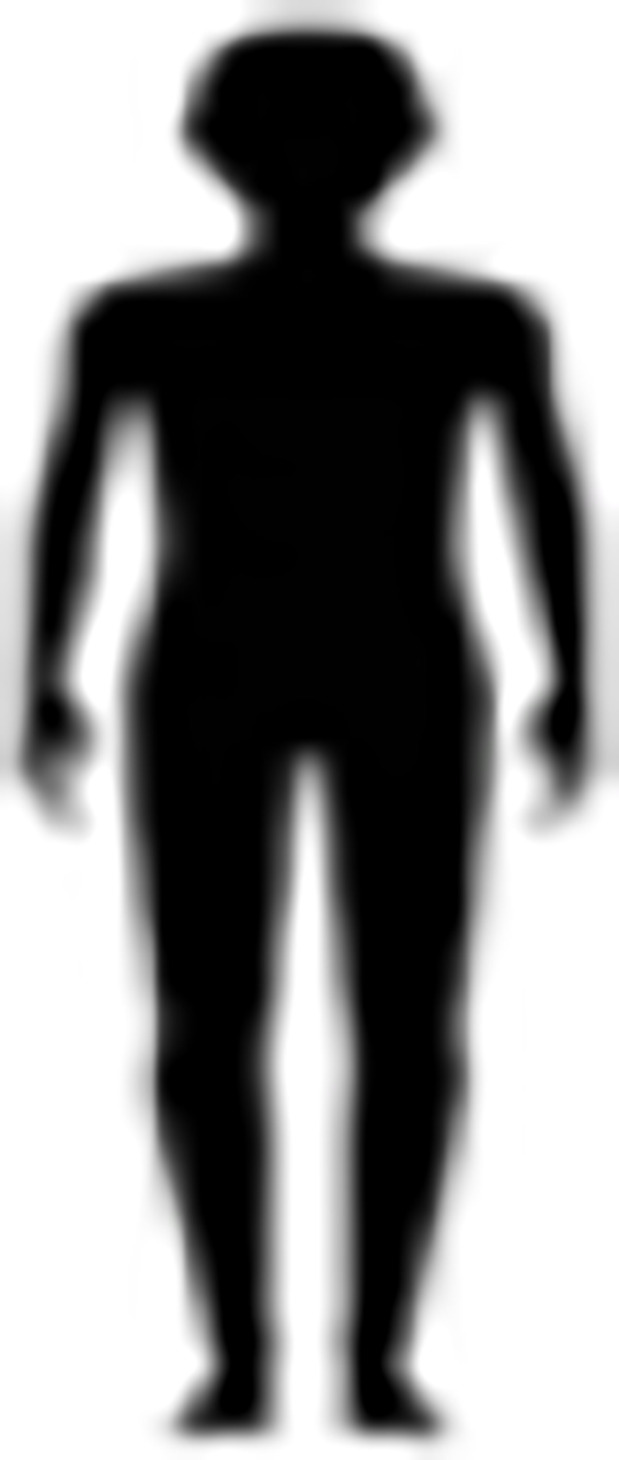	LLCMK2	HeLa	5:1	2h	18, 72hpi	Genome wide RNAi	AKT regulates intracellular *T.cruzi* replication (Caradonna et al., 2013)
Dm28c/TcII 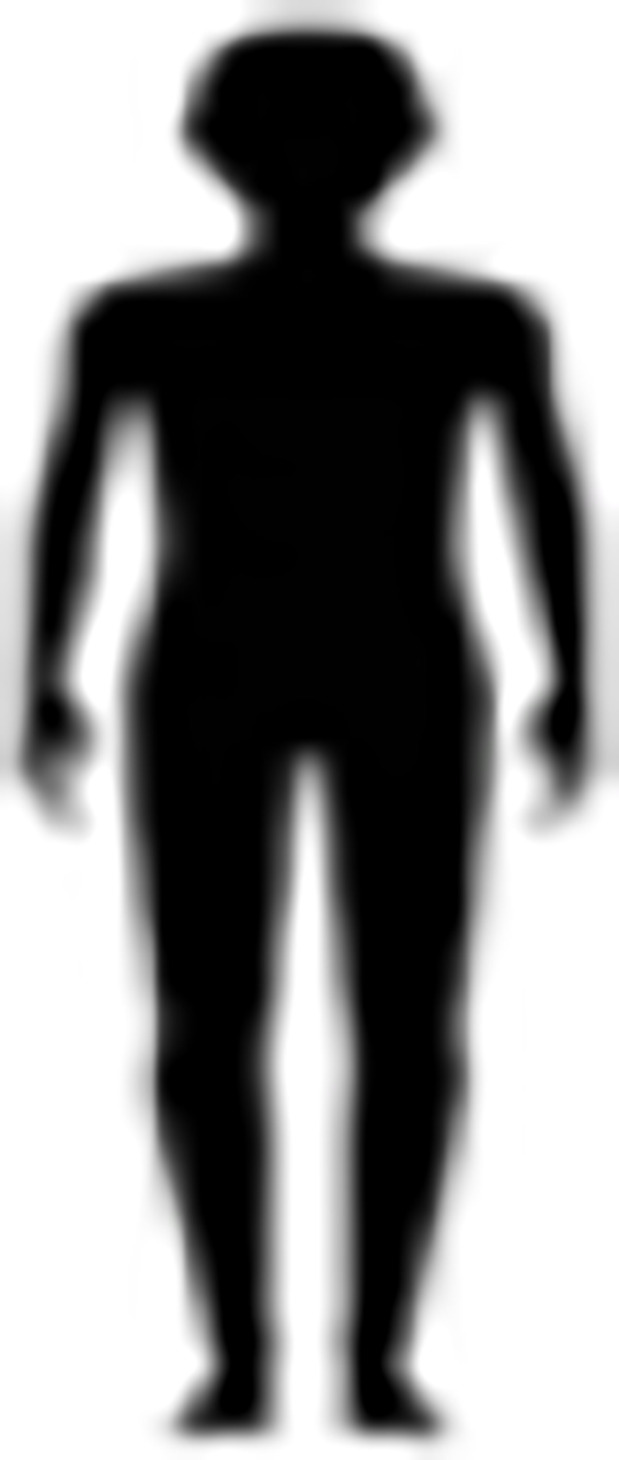	Vero	HeLa	10:1	4h	0, 3, 6hpi	Microarrays (Agilent)	Transcription upregulation of the PI3K/AKT pathway (Chiribao et al., 2014)
Dm28c/TcI 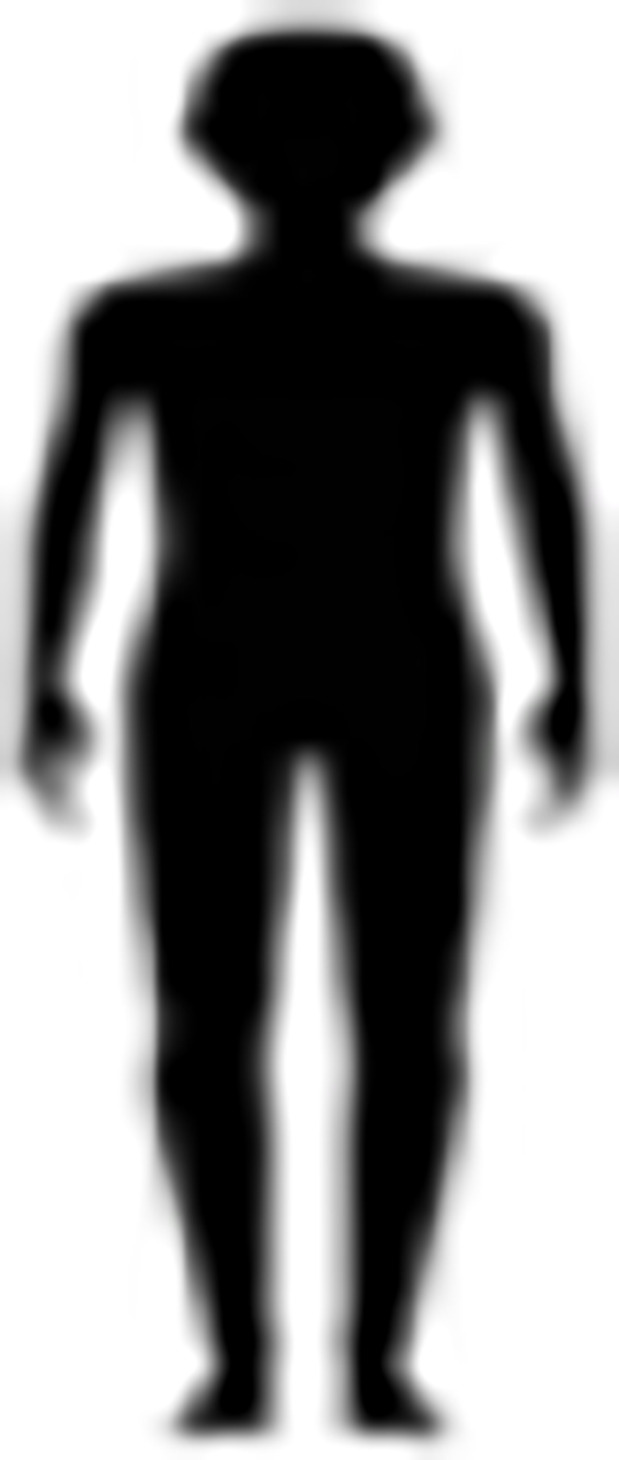	Vero	Primary human cardiomyocytes	10:1	2h	0, 2, 4, 6,12hpi	Microarrays (Agilent)/Western Blot	Transcription upregulation of the PI3K/AKT pathway. Overexpression of the phospho-Akt protein 24hpi (Libisch et al., 2018)
Y/TcII 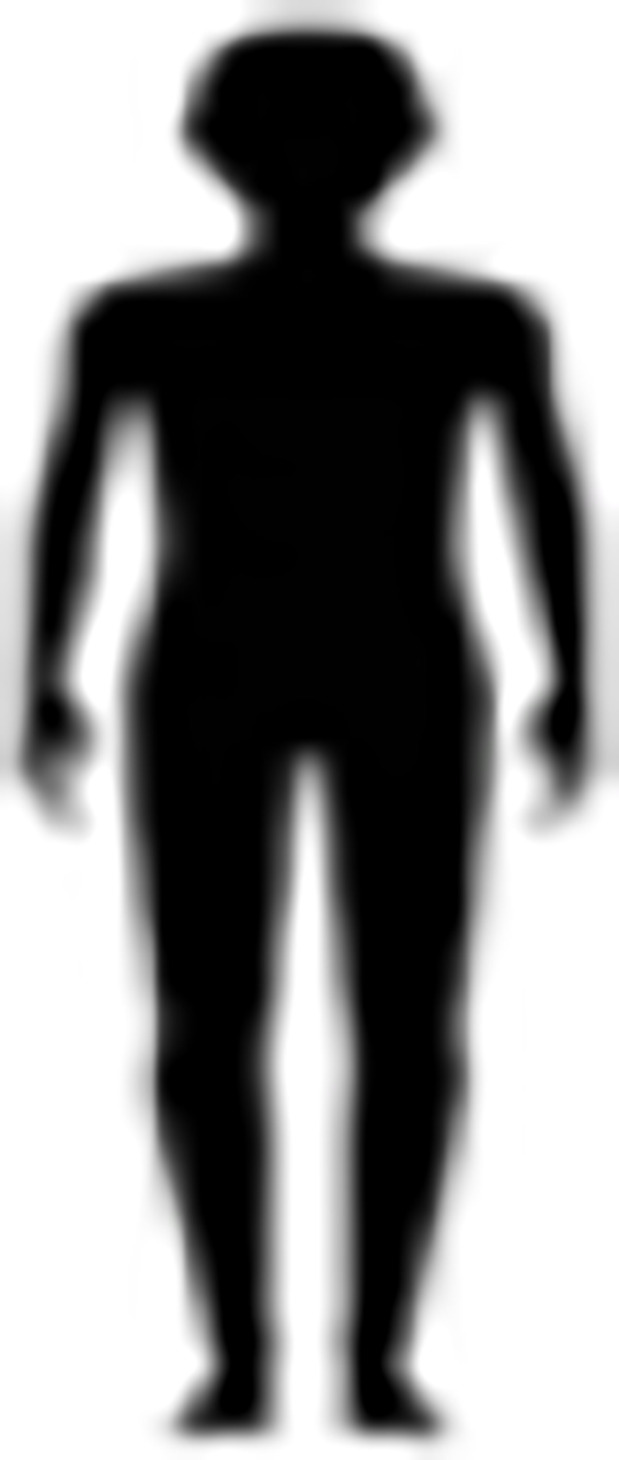 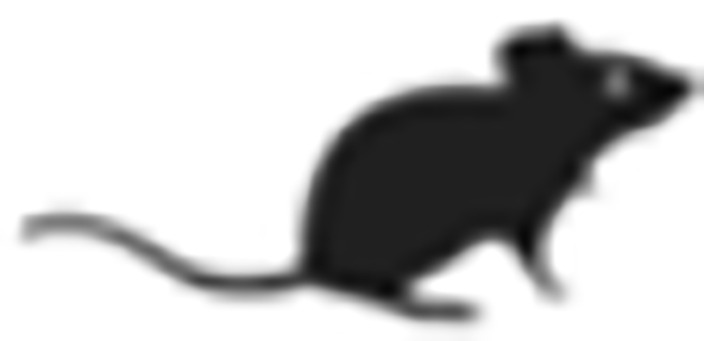	LLCMK2	C57BL/6 6-8 weeks mice, Heart tissue from CCC patients. *In vitro*: THP-1 cells	10^3^/ mouse 3:1 THP1	0	*In vivo*: 5 days *In vitro*: 48hpi	Microarrays (Agilent), Western blot	Transcription upregulation of the PI3Kγ pathway in CCC samples and hearts of infected mice. pAKT1 and pAKT2 were increased in the heart tissue from CCC patients. The PI3Kγ activation in myeloid cells was essential to restrict *T. cruzi* heart parasitism (Silva et al., 2018)

The orange color show those studies where an increase in transcriptomic (light color) and/or functional (dark color) activation of the PI3K/AKT pathway was reported. (PM, Propagation Model; MOI, Multiplicity of Infection; IT, interaction time; AT, analyzed time; ND, No Data available; LLCMK2, Kidney cells from Macaca mulatta; Vero, Kidney cells from Cercopithecus aethiops; L_6_E_9_, Myoblast from rat cells).

## Concluding Remarks

*T. cruzi*-host transcriptomic and functional studies give important clues to understand the changes suffered by the host cell in response to infection. In this review we analyzed how multiple experimental variables can significantly affect the final transcriptomic and functional responses, highlighting the difficulties to compare different works and drawing general conclusions to host-cell infection response. Concerning molecular mechanisms of pathogenesis in Chagas Disease, there are still many questions to answer, but the main point will be to select systems that could represent in the best possible way the human molecular characteristics of the disease, with studies comparable to each other. We found important differences in the OXPHOS response to *T. cruzi* infection depending on the origin of the infected cell (human or mouse), highlighting in the *in vitro* studies opposite responses depending on this variable, both at the transcriptomic and functional level. We caution about not considering the genetic and immunological differences between mice and humans before comparing results, and also, we believe that the use of *in vitro* models that include primary human cells, human induced pluripotent stem cells and 3D cultures (Breyner et al., 2020) are recommended options. Finally, delving into the activation mechanisms of PI3K/AKT would open new perspectives in host-directed therapies in Chagas disease.

## Author Contributions

MGL and CR contributed to conception and design of the study. MGL and NR organized the database. MGL wrote the first draft. All authors contributed to the article and approved the submitted version.

## Funding

This work was supported by: Research Council United Kingdom Grand Challenges Research Funder under grant agreement “A Global Network for Neglected Tropical Diseases” grant number MR/P027989/1; Agencia Nacional de Investigación e Innovación (UY) DCI-ALA/2011/023–502, “Contrato de apoyo a las políticas de innovación y cohesión territorial”, Fondo para la Convergencia Estructural del Mercado Común del Sur (FOCEM) 03/1.

## Conflict of Interest

The authors declare that the research was conducted in the absence of any commercial or financial relationships that could be construed as a potential conflict of interest.
